# Acute Myocarditis Masquerading as ST-Elevation Myocardial Infarction in a 17-Year-Old

**DOI:** 10.7759/cureus.29757

**Published:** 2022-09-29

**Authors:** Maham A Waheed, Cynthia Balasanmugam, Sergey Ayzenberg

**Affiliations:** 1 Internal Medicine, Maimonides Medical Center, Brooklyn, USA; 2 Cardiology, Maimonides Medical Center, Brooklyn, USA

**Keywords:** pericardium, cardiology research, cardiology imaging, myo-pericarditis, recurrent pericarditis, cardiology devices

## Abstract

Myocarditis can have a variable clinical presentation, ranging from asymptomatic to full-blown fulminant heart failure with severe left ventricular dysfunction or acute coronary syndrome (ACS) even ST-Elevation Myocardial Infarction (STEMI). Clinically myocarditis mimicking STEMI can present physicians with a great diagnostic challenge, especially in the absence of pro-dormal flu-like symptoms or a recent viral illness. Cardiac MRI has demonstrated superiority in detecting myocardial abnormalities and differentiating patients with myocarditis and from those with true STEMI.

## Introduction

Myocarditis is defined by the presence of inflammatory infiltrate in the myocardium with the presence of myocardial necrosis caused by viral infections, autoimmune disease, or cardiotoxic agents [1]. It can have a variable clinical presentation, ranging from asymptomatic to full-blown fulminant heart failure with severe left ventricular dysfunction [1,2] or acute coronary syndrome (ACS) even ST-elevation myocardial infarction (STEMI) [3].

Myocarditis mimicking MI is a rare clinical presentation with incidence reported to be 0.17 per 1000 man-years [4]. Moreover, clinically myocarditis mimicking STEMI can present physicians with a great diagnostic challenge, especially in the absence of pro-dormal flu-like symptoms. Given the non-specific patterns of presentation and the lack of specific diagnostic methods [5], such patients can often be misdiagnosed with ACS/STEMI. While endomyocardial biopsy has been recommended in guidelines [6], myocarditis is more often than not a clinical diagnosis based on patients’ medical history, clinical exam findings, laboratory studies, and imaging studies among which cardiovascular magnetic resonance (CMR) [7] has demonstrated superiority in detecting myocardial abnormalities and differentiating patients with myocarditis and from those with true STEMI. 

## Case presentation

A 17-year-old adolescent male with no significant past medical history presented to the emergency room with complaints of chest pain and tightness that started one hour prior to the presentation. He described the pain as sharp and achy, 9/10 in severity, radiating across the entire precordium and to both axillae. It was also associated with one episode of nausea and non-bilious, non-bloody vomiting. There was no related diaphoresis, lightheadedness, or dizziness. The patient also reported that the pain worsened with deep inspiration and was eventually relieved with ibuprofen.

Of note, he had a similar episode of pain the morning prior to presentation, which woke him up from sleep at 7 AM and persisted for 4 hours. During this initial episode, the pain was also relieved by ibuprofen. Overall patient reports he has an excellent functional capacity and denied any history of recent fevers, cough, or sick contacts. He denied any trauma to the chest or completing any strenuous work. Upon further history taking, there was no significant past medical or family history for cardiac disease or sudden cardiac death. The patient also denied any use of tobacco, illicit drugs, or alcohol use. 

On initial arrival at the emergency department (ED), the patient was afebrile and hemodynamically stable. On physical examination, the patient was not in any apparent distress. He was alert and oriented times three. Lungs were clear to auscultation bilaterally. The cardiac exam did not reveal any heart murmurs, JVD, or lower extremity edema. 

**Figure 1:**
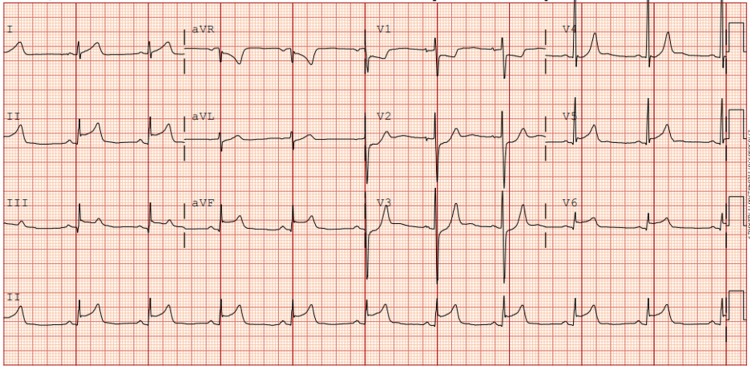
Admission EKG

The patient’s EKG on presentation revealed normal sinus rhythm with a heart rate (HR) of 61 beats per minute (bpm) with ST elevation >3 mm in leads II, III, aVF, and >1mm in V5 & V6. ST segment depression was noted in aVR, V1 to V4 (Figure 1). 

**Figure 2:**
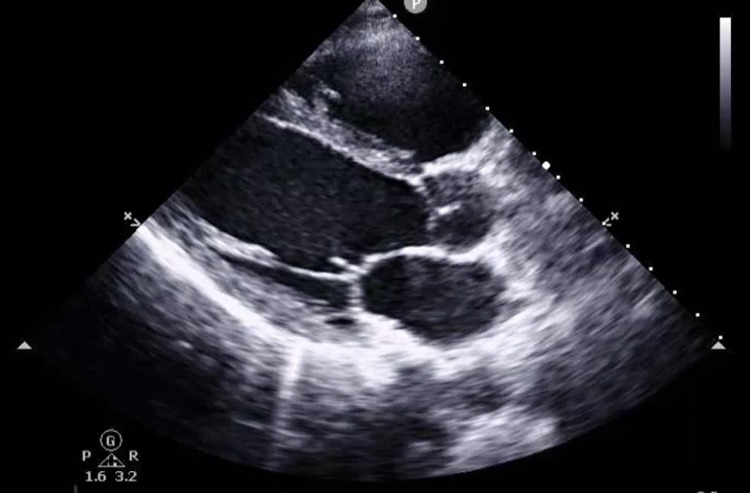
Parasternal long axis trans thoracic echocardiographic image

**Figure 3:**
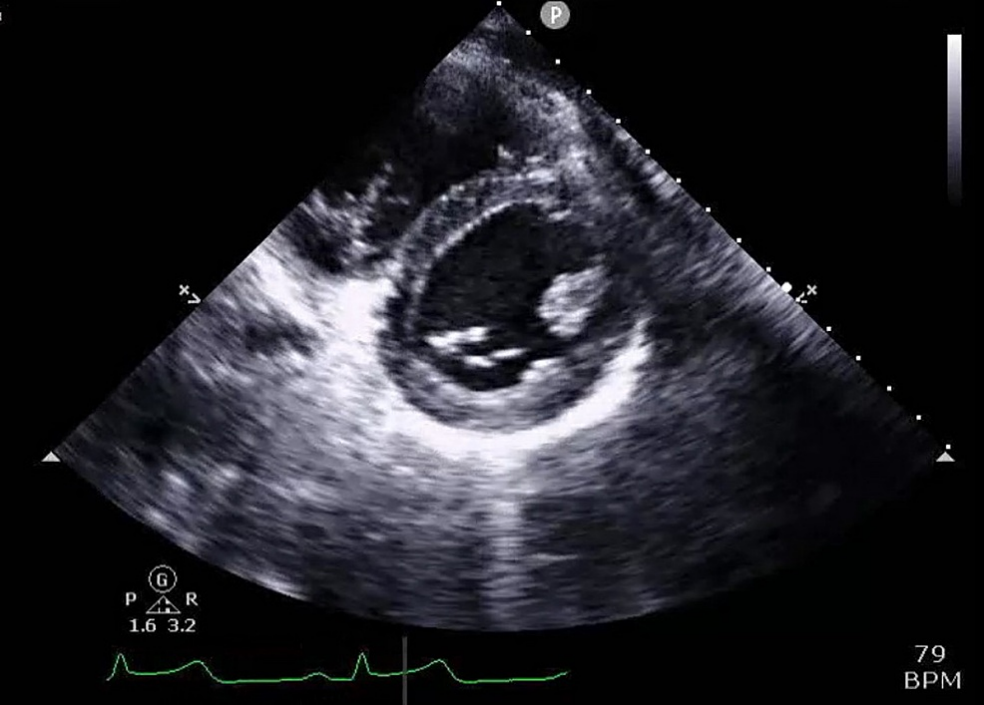
Parasternal short axis trans thoracic echocardiogram image

**Figure 4:**
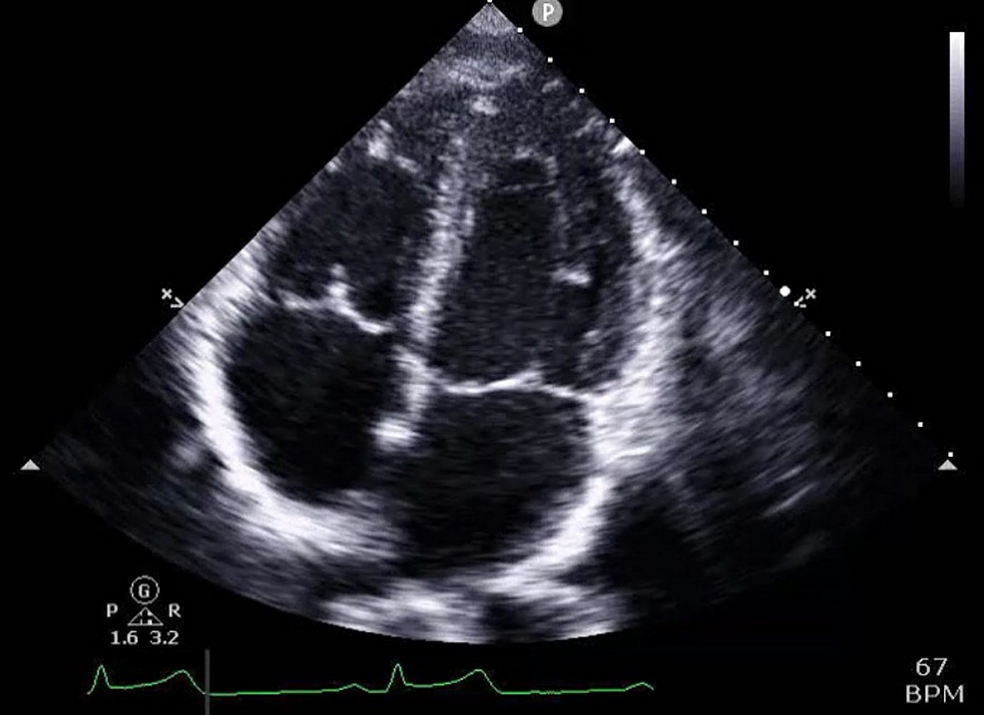
Apical view of the heart on trans thoracic echocardiogram

However, after administration of Ibuprofen, the pain subsided and the EKG almost nearly normalized except for subtle ST elevations in leads II, V5m V6. Laboratory results were significant for a peak elevated serum troponin of 95; BNP 56; D-dimer 173; negative respiratory viral panel. A chest X-ray was done and was unremarkable. Transthoracic echocardiography was done revealing a structurally normal heart with mild tricuspid valve insufficiency and no regional wall motion abnormalities (Figures 2-4).

**Figure 5:**
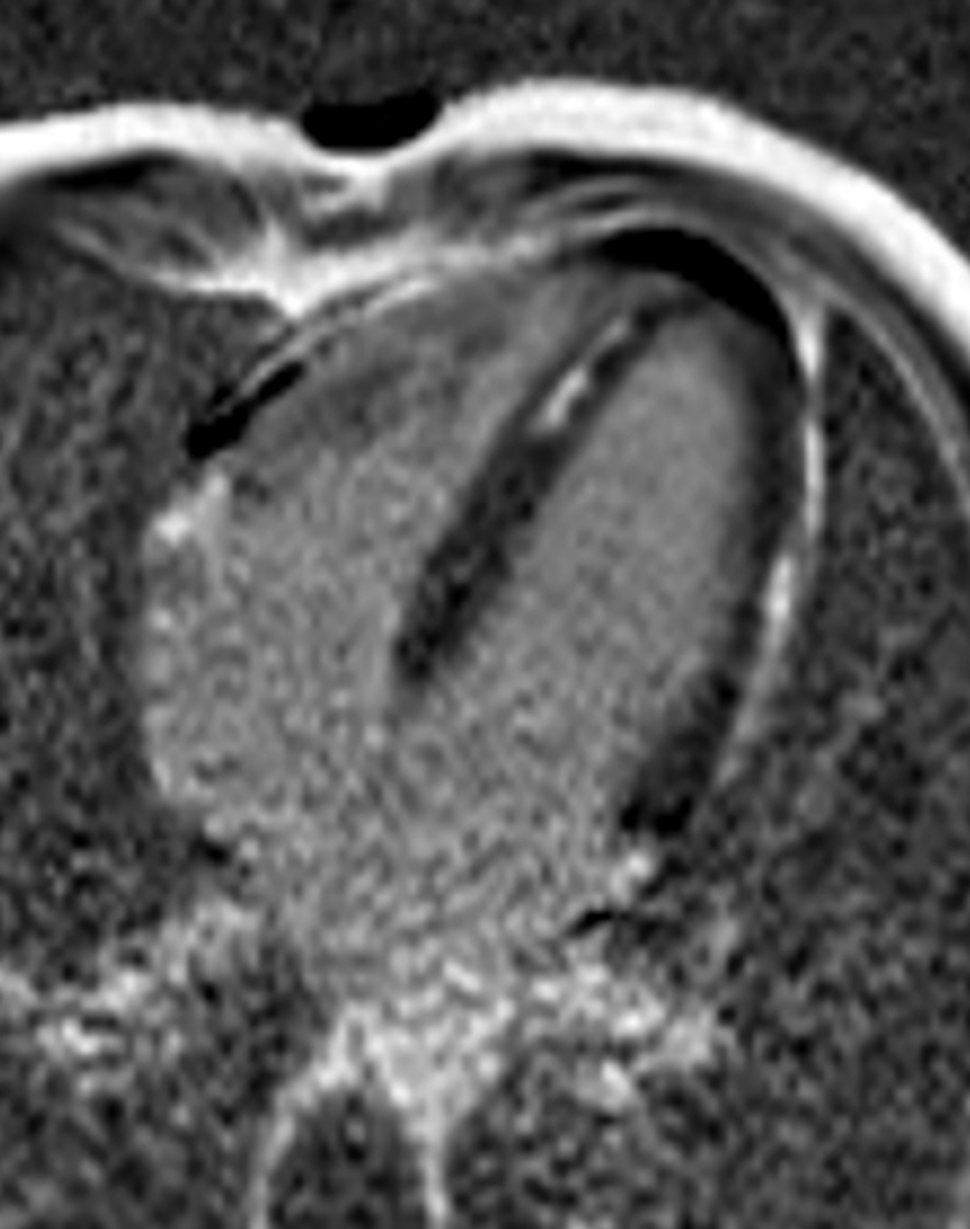
Cardiac MRI revealing myopericarditis

**Figure 6:**
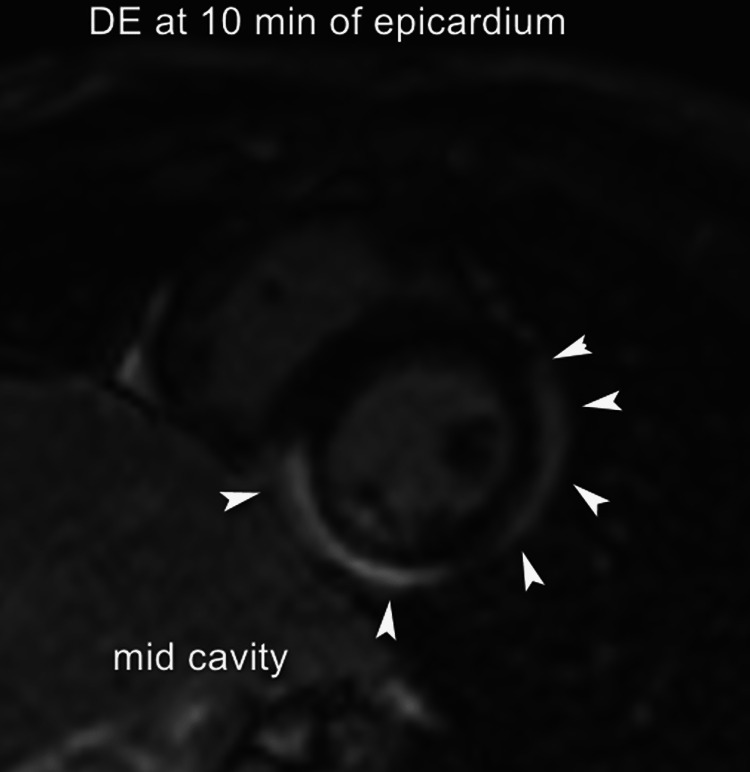
Cardiac MRI revealing mid-cavity inflammation

Cardiac MRI revealed extensive myopericarditis involving the mid cavity and apical region (Figures 5, 6).

The patient was discharged after two days of hospitalization. He had multiple follow-up echocardiograms done as an outpatient which revealed no development of wall motion abnormalities, changes in ejection fraction (EF), or structural heart disease. No further pain episodes were reported.

## Discussion

Here we present the case of a young man without any flu-like symptoms who presented to the ED with classic ACS presentation and was found to have acute myocarditis masquerading as STEMI. When evaluating patients with chest pain, it is vital to rule out ACS on initial evaluation.

In patients with true acute myocardial infarction and ST-segment elevation on EKG, early reperfusion therapy provides the most benefit. Therefore, since “time is myocardium”, it is vital to diagnose patients with ACS as soon as possible. However, it is equally important to not misdiagnose or confuse other causes of ST-segment elevation with acute myocardial infarction [8,9]. Patients with myocarditis mimicking STEMI presentation often present great diagnostic as well as therapeutic challenges and may mistakenly undergo unnecessary interventions such as thrombolytic therapy or urgent coronary angiography [10-13]. Studies done to evaluate inappropriate thrombolytic use in patients with suspected ACS discovered that 10 of 93 patients (11%) in one study [14] and 35 of 609 patients (5.7%) in another study [15], did not have an infarction. 

It has been recommended that a thorough clinical evaluation comprising of wholesome history and physical exam findings is paramount in helping diagnostic evaluation in these patients [3,12,16]. Although some case reports have described nonrheumatic streptococcal myocarditis to present as a STEMI mimic [12,13] it is important to recognize that regardless of underlying viral etiology, myocarditis can present similar to STEMI or STEMI-like syndrome and the use of multimodality imaging may aid in diagnosis. Cardiac MRI (CMR) allows for optimal differentiation between normal and diseased myocardial tissue via gadolinium-based contrast agents and has been used as a non-invasive measure for diagnosing myocarditis [16,17]. In acute myocardial infarction (AMI), late gadolinium enhancement (LGE) usually reveals subendocardial or transmural enhancement with edema localized to the culprit vessel whereas in myocarditis the edema may be segmental or diffused [16].

## Conclusions

Cardiac MRI (CMR) allows for optimal differentiation between normal and diseased myocardial tissue via gadolinium-based contrast agents and has been used as a non-invasive measure for diagnosing myocarditis. The use of multimodality imaging is vital in diagnosing acute myopericarditis and should be employed during the work-up of acute chest pain in young adolescents who may present with acute chest pain mimicking STEMI-like clinical presentations.
